# *FAAH* polymorphism (rs324420) modulates extinction recall in healthy humans: an fMRI study

**DOI:** 10.1007/s00406-021-01367-4

**Published:** 2021-12-10

**Authors:** Jennifer Spohrs, Martin Ulrich, Georg Grön, Paul L. Plener, Birgit Abler

**Affiliations:** 1 Department of Child and Adolescent Psychiatry/Psychotherapy, Ulm University, Ulm, Germany; 2 Clinic for Psychiatry and Psychotherapy III, Ulm University, Ulm, Germany; 3 Department of Child and Adolescent Psychiatry, Medical University Vienna, Vienna, Austria

**Keywords:** Endocannabinoid system, Anandamide, Anxiety disorders, FAAH rs324420, Extinction recall

## Abstract

Gold standard treatments for anxiety- and trauma-related disorders focus on exposure therapy promoting extinction learning and extinction retention. However, its efficacy is limited. Preclinical and particularly animal research has been able to demonstrate that homozygosity for the fatty acid amide hydrolase (*FAAH)* C385A allele, similar to FAAH inhibition, is associated with elevated concentrations of anandamide (AEA) and facilitates extinction learning and extinction recall. However, in humans, the underlying neurobiological processes are less well understood, and further knowledge might enhance the development of more effective therapies. In this functional magnetic resonance imaging (fMRI) study, a fear conditioning, fear extinction and extinction recall paradigm was conducted with 55 healthy male adults. They were genotyped for the *FAAH* single-nucleotide polymorphism (SNP) rs324420 to investigate differences related to extinction recall in neural activation and State–Trait Anxiety Inventory (STAI) ratings between AC heterozygotes and CC homozygotes (*FAAH* C385A SNP). Differential brain activation upon an unextinguished relative to an extinguished stimulus, was greater in AC heterozygotes as compared to CC homozygotes in core neural structures previously related to extinction recall, such as the medial superior frontal gyrus, the dorsal anterior cingulate and the anterior and middle insular cortex. Furthermore, AC heterozygotes displayed higher AEA levels and lower STAI-state ratings. Our data can be interpreted in line with previous suggestions of more successful extinction recall in A-allele carriers with elevated AEA levels. Data corroborate the hypothesis that the endocannabinoid system, particularly AEA, plays a modulatory role in the extinction of aversive memory.

**Supplementary Information:**

The online version contains supplementary material available at 10.1007/s00406-021-01367-4.

## Introduction

The past decades have seen major advances in the understanding and treatment of stress and anxiety disorders, such as posttraumatic stress disorder (PTSD), panic disorder or phobias, which are highly frequent and debilitating psychiatric disorders [1]. Current treatment options, such as psychopharmacological treatments and exposure-based psychotherapy, have been demonstrated to be effective; however, they leave room for improvement [2]. To further advance available treatment options, neuromodulatory, genetic, physiological and behavioral processes involved in stress- and anxiety disorders have been intensely studied and have expanded knowledge regarding underlying processes. Among others, particularly, the endocannabinoid system (ECS) has become a subject of interest [3, 4] as previous research suggests a modulating role in stress- and anxiety disorders, especially in balancing the homeostasis after stress. Accordingly, altered endocannabinoid levels were identified in stress-related disorders, such as PTSD [5].

The plant *Cannabis sativa* has been consumed for its anxiolytic effects for thousands of years. Recently, major research advances were achieved regarding the endocannabinoid system, with its well-known pharmacological accessibility [6]. Research supports that particularly modulation of anandamide (AEA) transmission, one of the main endocannabinoids, could have beneficial effects in the treatment of anxiety. Accumulating evidence indicates that the genetic single-nucleotide polymorphism (SNP) rs324420 (C385A) in the fatty acid amide hydrolase (*FAAH*) gene, coding for the AEA degrading enzyme, modulates processes associated with extinction learning [7, 8]. Extinction learning is essential for effective exposure therapies in clinical settings and its efficacy can be tested by extinction recall checks [9]. Approximately 38% of the European population are carriers of the A-allele (33% AC heterozygotes, 5% AA homozygotes) for the rs324420 SNP, leading to the FAAH enzyme being more vulnerable to proteolytic degradation. Consequently, in AA homozygotes the expression of FAAH activity is assumed to be less than half of the expression of FAAH activity of wildtype lymphocytes leading to higher peripheral AEA levels in AA/AC allele carriers [7, 8, 10, 11]. In line with these findings, animal studies were able to demonstrate enhanced extinction processes when using cannabinoid agonists or FAAH inhibitors [12–14]. Studies point towards beneficial effects for human carriers of the homozygote AA and the heterozygote AC type regarding anxiety processing [7, 15, 16]. Recently, Mayo et al. [8] have found that gene-dependent higher levels of AEA in humans enhanced fear extinction and extinction recall, and that elevated AEA levels in AA homozygous carriers have a protective function during stress-related responses, in a way that AA homozygotes showed no significant decreases in AEA levels after a stress task. However, this effect was not observed for AC heterozygotes [8].

With regard to the neurobiological basis, Hariri et al. [15] found that genetic differences in endocannabinoid signaling in humans modulated threat- and reward-related brain function. A-allele carriers (*FAAH* C385A SNP) showed lower amygdala activation in a threat-related face processing task and increased activation in the ventral striatum in a reward task [15]. In addition, in their functional magnetic resonance imaging (fMRI) study, Gunduz-Cinar et al. [17] observed that carriers of the A-variant of the allele presented significantly enhanced habituation to threatening faces, a process that was mediated by amygdala activity [17]. In another fMRI study with healthy humans [18], A-allele carriers displayed enhanced frontal cortex–amygdala connectivity during rest, but no differences in emotion regulation strategies during a paradigm with negative pictures [18]. Furthermore, Zabik et al. [19] showed genotype-dependent differential activation in brain regions related to fear conditioning and extinction using an associative learning task, however without effects on the behavioral level [19]. Combined, the A-allele of the SNP rs324420 has been linked to modulate pain perception [20], lower anxiety [7], and enhanced fear extinction learning [8].

To investigate the mechanisms of fear learning, the most frequently applied paradigm is based on Pavlovian fear conditioning, where an initially neutral stimulus (CS^−^) is presented with an unconditioned aversive stimulus (US). After multiple presentations, the neutral stimulus elicits the fear response even in absence of the aversive stimulus, and is, thus, referred to as the conditioned stimulus (CS^+^). For subsequent fear extinction learning, this conditioned stimulus is presented multiple times, which leads to the deletion or reduction of the fear response (extinguished stimulus, CS^+extinguished^). To test fear extinction learning, the stimuli are presented during extinction recall after fear extinction consolidation [9]. Here, the stimulus is once again presented and the magnitude of the fear response is compared to either another conditioned stimulus (unextinguished stimulus, CS^+unextinguished^), which was not displayed during fear extinction learning, or to another neutral stimulus (CS^−^) [21]. These paradigms have been highly beneficial to facilitate the understanding of the underlying mechanism during fear learning processes. Regarding the neural networks involved in extinction recall, a recent meta-analysis by Fullana et al. [21] summarized the left anterior and right dorsolateral prefrontal cortex, dorsal aspects of the anterior cingulate, bilateral anterior insular cortex, left parietal operculum and right anterior hippocampus from studies comparing neural activation related to the extinguished stimulus with that related to the unextinguished stimulus.

Based on what is known so far from rodent studies about the role of the *FAAH* C385A SNP in extinction recall, the present study was designed to investigate its allele-associated effects on the neural signatures of extinction recall in humans. Therefore, 55 healthy male subjects underwent successful Pavlovian-like fear conditioning and fear extinction (see [22]). Extinction recall was then investigated with functional magnetic resonance imaging. We predicted differential neural activation upon an unextinguished relative to an extinguished stimulus, to be greater in AC heterozygotes as compared to CC homozygotes in regions associated with extinction recall as identified by Fullana et al. ([21]; see above). Behaviorally, carriers of the A-allele of the *FAAH* C385 SNP as compared to the CC homozygotes were expected to demonstrate lower anxiety ratings as measured by means of task-related subjective ratings of the State-Trait Anxiety Inventory (STAI), and similarly, based on the findings of previous studies [7], we also predicted A-allele carriers to display lower anxiety ratings than CC individuals on the trait scale.

## Materials and methods

### Participants

55 right-handed, male subjects participated in the study (mean age = 22.8 years, SD = 3.0 years). They were included after completing a screening procedure to exclude confounding medical or psychiatric conditions and substance use (see Supplementary Information). Four participants had to be excluded due to acute intake of medication and technical failures. All participants signed an informed consent prior to the study, which was conducted according to the guidelines of the Declaration of Helsinki as approved by the Ethics Board of Ulm University, Germany.

### Experimental task during fMRI

**Fig. 1:**
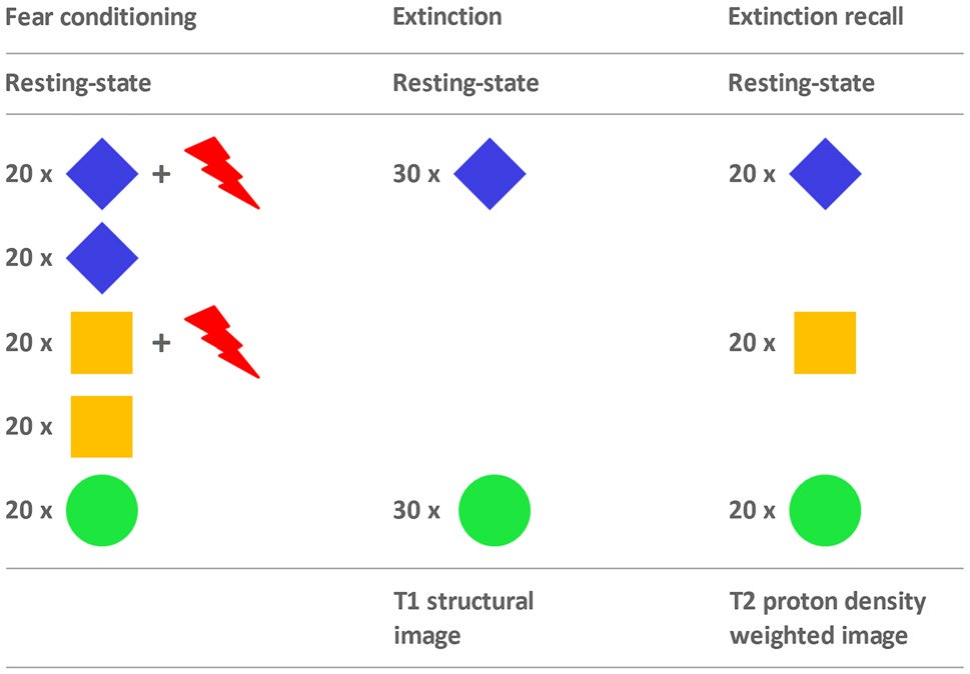
Schematic overview of the experimental setup over 3 days. Blue and yellow depict the conditioned stimuli (CS^+a^ and CS^+b^), green the neutral stimulus (CS^−^), and the red arrows represent the unconditioned, unpleasant stimulus (US), heat, applied with a thermal stimulation device on the right shinbone. During fear conditioning (day 1), 20 stimuli of each condition were presented (CS^−^, CS^+a^, CS^+b^, CS^+a^ + US, CS^+b^ + US) at a reinforcement rate of 50% in the case of CS^+^  + US. During fear extinction (day 2), CS^+a^ and CS^−^ were presented 30 times each. During extinction recall (day 3), the conditions CS^−^, CS^+a (extinguished)^, and CS^+b (unextinguished)^ were presented 20 times each

Participants completed a classical Pavlovian fear conditioning, fear extinction, extinction recall paradigm over 3 days (see Fig. 1), which was based on previous experiments [9]. On each day, participants were enrolled between 7 and 9 AM and were instructed to fast overnight, with no breakfast and only water allowed before the experiment to metabolically control AEA levels [3, 23]. During fear conditioning, three different geometric stimuli were presented on a computer screen. One stimulus remained neutral (CS^−^) and two stimuli (CS^+a^, CS^+b^) were coupled with unpleasant thermal stimulation (US) to the right shinbone at a partial reinforcement rate of 50% (for more details please see Supplementary Information). All stimuli (CS^−^, CS^+a^, CS^+b^, CS^+a^ + US, CS^+b^ + US) were presented 20 times in pseudo-randomized order. To avoid biasing by effects of color or shape, shapes and colors of stimuli were balanced across subjects. In the extinction phase, CS^−^ and CS^+a^ were presented 30 times each, without presentation of the US. To test extinction recall, all three stimuli (CS^−^, CS^+a^, CS^+b^) were presented 20 times without the US. On all three experimental phases, participants were informed that thermal stimulation was possible. To program and present the trials, Presentation 14.8 (Neurobehavioral Systems Inc., Albany, CA) was used. The stimuli were presented to the participants on a 32″ liquid crystal display (LCD) (NordicNeuroLab AS, Bergen, Norway) at a resolution of 1280 × 720 pixels, which was installed behind the MR scanner and was visible by means of a double-mirror mounted on top of the head coil. Thermal stimulation was carried out by means of an MRI-compatible ATS thermode (30 × 30 mm, TSA-II, Medoc Advanced Medical Systems, Ramat Yishai, Israel).

### Blood sampling and FAAH genotyping

At the very beginning of the entire experimental regimen, 2 × 7.5 ml EDTA blood samples were obtained from the participants. Deoxyribonucleic acid (DNA) was extracted applying standard protocols of a commercial extraction kit (MagNA Pure 96 DNA and Viral NA Small Volume Kit) and the MagNA Pure 96 (Roche Diagnostics, Mannheim, Germany). Genotyping at *FAAH* C385A was performed via real-time quantitative polymerase chain reaction using melting curve detection analysis with Cobas z480 Analyser (LightCycler) (Roche Diagnostics, Mannheim, Germany). The primers were obtained from TIB Molbiol (Berlin, Germany).

### Fear and anxiety ratings

Using a visual analog scale and an MRI-compatible trackball (NAtA TECHNOLOGIES, Coquitlam, Canada), participants were instructed to rate the subjectively perceived fear related to each stimulus before and after the fMRI experiment *(How afraid are you of the stimulus coupled to this symbol?* [9]*.* The scale ends were defined as *0 not at all* and *10 very much.* Ratings were performed while inside the MRI scanner, directly before and after the experiment. Additionally*,* participants completed the state version of the State-Trait Anxiety Inventory [24] before and after the fMRI session outside the scanner, within 10 min pre- or post-scan, respectively. Unfortunately, STAI scales were only partially completed in some cases so that data from 28 CC and from 12 AC individuals were entered the statistical comparison of pre/post rating data below. The trait version of the State–Trait Anxiety Inventory was filled out by all but one participant before the start of the entire experimental regimen. Previous studies have suggested that A-allele carriers present lower anxiety scores [7]. Thus, scores were used to investigate if healthy participants differ in their subjective fear and STAI anxiety ratings, with the assumption that A-allele carriers display lower anxiety ratings than CC individuals.

### MRI data acquisition

Acquisition of magnetic resonance imaging data was performed on a 3 Tesla MAGNETOM Prisma (Siemens AG, Erlangen, Germany) with a 64-channel head/neck coil. For estimation of task-related brain activation, the T2*-weighted blood oxygen level dependent (BOLD) signal was measured using echo-planar imaging with the following parameters: TR = 2000 ms, TE = 33 ms, flip angle = 90°, bandwidth = 2136 Hz/Px, PAT factor = 2 (GRAPPA mode), FOV = 220 mm, matrix size = 90 × 90, number of slices: 32, slice orientation: transversal, acquisition: ascending, slice thickness: 3.0 mm, interslice gap = 1.0 mm, voxel size: 2.44 mm × 2.44 mm × 4.00 mm. During the fMRI session 432 EPI volumes were acquired (scan time: 14.3 min).

At the participants’ second visit, and after completion of the main experimental task, a high-resolution T1-weighted structural image was obtained by administering a 3D magnetisation prepared rapid acquisition gradient echo sequence (MPRAGE; TR = 2300 ms, TE = 2.98 ms, inversion time = 900 ms, flip angle = 9°, FOV = 256 mm, matrix size: 256 × 256, voxel volume = 1 mm^3^, slice orientation: sagittal; PAT factor = 2 (GRAPPA mode); scan time = 5.21 min).

### Whole brain analysis of fMRI data

Image pre-processing and analyses were performed using SPM12 according to the standard procedures (see Supplementary Information). A detailed description of the analyses for fear conditioning and fear extinction is reported in Spohrs et al. [22]. Per participant, pre-processed task-based fMRI data were modeled using a single-session-separated general linear model. Extinction recall was modeled with three regressors representing the first three trials of CS^+a (extinguished)^, CS^+b (unextinguished)^, and CS-, respectively [9, 21]. Three further regressors of no interest represented trials 4–20 associated with each condition. Trial onsets were convolved with the canonical hemodynamic response function (HRF) and its temporal derivative. Data were high-pass filtered (frequency cut-off: 1/128 s) to remove low-frequency scanner drifts. An autoregression model of polynomial order 1 was used to account for temporally correlated residual errors. After model estimation, contrast images representing estimated activation for each regressor versus baseline were computed. The contrast images representing the first three trials of CS^+a (extinguished)^ and CS^+b (unextinguished)^ were collected from all participants and subjected to a second-level random-effects analysis, implemented in SPM12 as a flexible factorial design with the factors *subject, genotype* (*FAAH*) and *condition*. To investigate neural signaling related to extinction recall irrespective of genotype, an average *t*-contrast across both genotype groups was set up (CS^+b (unextinguished)^ > CS^+a (extinguished)^). Next, addressing the prediction of higher neural extinction recall in AC heterozygotes relative to CC homozygotes, the following *t*-contrast was set up, modeling the direction of the genotype × condition interaction of interest: (CS^+b (unextinguished)^_AC_ > CS^+a (extinguished)^_AC_) > (CS^+b (unextinguished)^_CC_ > CS^+a (extinguished)^_CC_). The thresholds for the resulting statistical parametric maps were set at a voxel-height level of *p* < 0.001, uncorrected, and an FWE-corrected cluster threshold of *p* < 0.05 corresponding to 173 contiguously significant voxels.

### Analysis of fear and anxiety ratings

Using STATISTICA13 (TIBCO Software Inc., Palo Alto, CA, USA), two separate analyses of variance (ANOVA) for repeated measures were computed to test the hypothesis that A-allele carriers present lower anxiety levels with regard to the subjective fear ratings and the STAI-state anxiety scores.

## Results

### FAAH C385A SNP genotyping

*FAAH* C385A SNP genotyping revealed 17 AC heterozygous and 34 CC homozygous individuals. Consistent with the rare distribution of AA homozygotes in the population, none of the participants carried this allele combination. As previously reported [22], mean plasma levels of AEA obtained from blood samples taken before the entire experimental regimen were significantly (*t* (49) = 2.81, *p* = 0.007) higher in AC heterozygotes ([AEA]_AC_ = 0.49 ± 0.16 pmol/ml) compared to the individuals homozygous for the C-allele ([AEA]_CC_ = 0.38 ± 0.13 pmol/ml) (Cohen’s *d* = 0.755).

### Neuroimaging data

#### FAAH C385A SNP whole-brain group differences and neural extinction recall signaling

At the given significance level, for the entire sample, no clusters were found for the main effect of extinction recall. At an uncorrected level (*p* < 0.001), this contrast revealed one cluster in the left anterior insula that did not, however, survive correction for multiple comparisons (cluster size: 38 voxels; peak coordinates: − 36, 28, 4; *z*-score: 4.73).

**Fig. 2:**
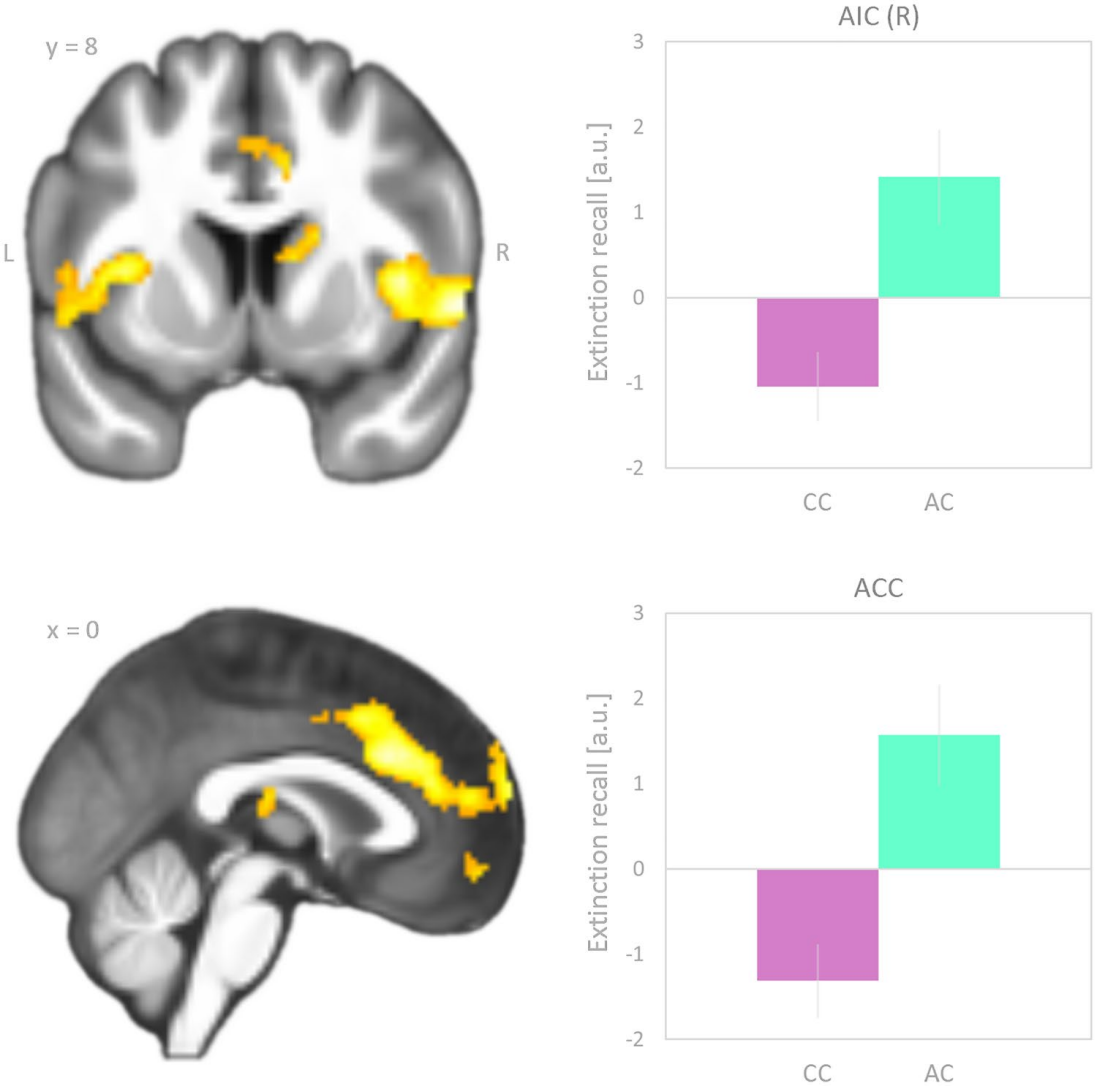
Brain regions where carriers of the *FAAH* A-allele, relative to CC homozygotes, displayed a significantly greater effect of extinction recall, defined as the difference “CS^+b (unextinguished)^ minus CS^+a (extinguished)^”. As previously introduced [21, 41, 42], a positive going contrast value indicates extinction recall, while a negative going contrast value indicates the reverse. This is exemplified by the bar graphs on the right side of the figure, for two representative brain regions, the right anterior insula (AIC) and the anterior cingulate cortex (ACC). Significance was assessed at *p* < 0.001 (voxel level) and *p* < 0.05 (cluster level FWE corrected, corresponding to 173 voxels). The statistical parametric map was superimposed on coronal and sagittal sections of the group averaged T1 image. Coordinates refer to Montreal Neurological Institute (MNI) space. Differential parameter estimates were extracted from the right anterior insula (869 voxels; upper right panel) and the anterior cingulate cortex (2341 voxels; lower right panel). *a.u.* arbitrary unit; *L* left; *R* right

Between-groups contrasting showed that AC heterozygotes relative to CC homozygotes had significantly greater differential neural activation related to extinction recall in the following brain regions (Fig. 2 and Table 1): bilateral anterior and middle cingulate cortex, bilateral anterior and middle insular cortex, bilateral superior temporal gyrus, right Rolandic operculum, left middle temporal gyrus, left postcentral gyrus, and right caudate nucleus (whole-brain analysis, voxel level *p* < 0.001, cluster level *p* < 0.05, FWE corrected, corresponding to 173 voxels).

**Table 1:** Brain regions showing a higher signal (CS^+b (unextinguished)^ > CS^+a^ ^(extinguished)^) related to extinction recall in the FAAH AC heterozygotes as compared to the CC homozygotes

	Brain region	Number of voxels	Peak voxel (MNI space)			
			*x*	*y*	*z*	*z*-score
L	Medial superior frontal gyrus	2341	− 6	62	22	4.58
	Dorsal anterior cingulate		0	24	30	4.45
R	Dorsal anterior cingulate		4	40	22	4.32
L	Anterior insula	303	− 34	6	8	4.29
L	Middle insula		− 42	2	− 2	3.58
R	Anterior insula	869	40	12	6	4.69
R	Middle insula		48	2	0	4.15
R	Superior temporal pole		58	6	0	4.67
L	Superior temporal gyrus	278	− 62	− 14	4	4.27
L	Middle temporal gyrus		− 52	− 30	4	4.13
L	Postcentral gyrus	287	− 60	− 18	32	4.56
L	Postcentral gyrus		− 48	− 22	46	3.86
R	Caudate nucleus	506	12	4	14	4.36
R	Caudate nucleus		20	22	2	3.88

*n* = 51

Significance: *p* < 0.001 (voxel level) and FWE-corrected (*p* < 0.05) cluster sizes corresponding to 173 contiguous voxels, whole-brain analysis

*L* left; *R* right. *MNI* Montreal Neurological Institute

#### Subjective fear ratings

Subjective fear ratings confirmed the validity of the conditioning–extinction–extinction recall paradigm. For data regarding fear conditioning, please refer to Spohrs et al. [22]. As a marker for successful extinction recall, the difference between ratings for the CS^+a (extinguished)^ pre (*M* = 5.08, SD = 2.88) and the CS^+b (unextinguished)^ pre (*M* = 5.78, SD = 3.12) was significant (*t* (50) = 1.98, *p* = 0.027, one-tailed). The difference between the CS^+a (extinguished)^ pre and the neutral stimulus CS^−^ pre (*M* = 0.98, SD = 1.61) was also significant (*t* (50) = 9.97, *p* < 0.001). When looking at the post experimental differences between the extinguished stimulus (CS^+a (extinguished)^ post: *M* = 2.72, SD = 2.68) and the neutral stimulus (CS^−^ post: *M* = 0.52, SD = 1.34), these differences remain significant after extinction recall (t (50) = 5.38, *p* < 0.001). The same holds for the unextinguished stimulus (CS^+b (unextinguished)^ post: *M* = 2.98, SD = 2.60) and the neutral stimulus (*t* (50) = 6.03, *p* < 0.001). To check for differences between the *FAAH* genotype (AC vs. CC individuals), a repeated-measures ANOVA with the factors *time point* (pre, post), *genotype* and *stimulus,* was performed, which revealed no significant main effect for *genotype* (*F* (1,49) = 0.18, *p* = 0.669), while main factors *time point* (*F* (1,49) = 67.75, *p* < 0.001) and *stimulus* (*F* (2,98) = 53.98, *p* < 0.001) were significant as expected. However, there was no significant interaction of all three main factors *time point* by *stimulus* by *genotype* (*F* (2,98) = 2.07, *p* = 0.131).

#### STAI-S and STAI-T

**Fig. 3:**
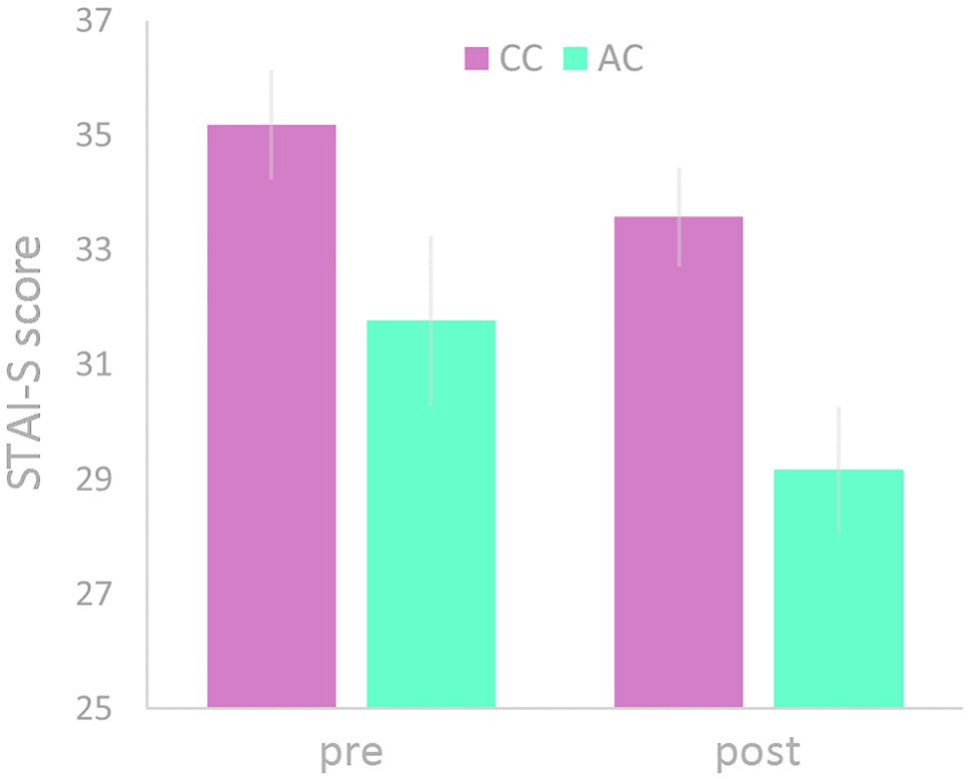
Genotype-related group differences in the state version of the State–Trait Anxiety Inventory (STAI-S) before (pre) and after (post) the extinction recall fMRI experiment. Bars represent group averages; error bars denote the standard error of the mean

Comparison of state anxiety ratings (STAI-S) between CC and AC individuals before the extinction recall did not show a statistically significant group difference (*t* (38) = 1.94, *p* = 0.059), while average rating scores of the CC group (mean = 35.2 ± 5.1) were numerically higher than those of the AC group (mean = 31.8 ± 5.2) (Cohen’s *d* = 0.686). After the extinction recall fMRI session, STAI-S ratings differed significantly (*t* (38) = 2.92, *p* = 0.006) between CC (mean = 33.6 ± 4.6) and AC (mean = 29.2 ± 3.8) individuals (Cohen’s *d* = 1.01) (Fig. 3). Assuming that the numerical group difference before the extinction recall fMRI experiment might have conditioned the group difference afterwards, the test for group differences was repeated using a repeated-measures ANCOVA where individual state anxiety ratings before the experiment entered the analysis as covariate to compensate for these numerical differences at this time point (baseline correction). This computation revealed indeed a significant *time point* by covariate interaction (*F*(1,37) = 15.65, *p* < 0.001), while the interaction of both main factors *time point* and *genotype* remained significant (*F*(1,37) = 4.23, *p* = 0.047), indicating that the pre-to-post decrease in state anxiety ratings of the AC group (2.6 score units) was significantly greater than that of the CC group (1.6 score units) despite the numerical group difference before the experiment.

For the trait version of the STAI, a two-sample *t*-test did not reveal a significant difference between *FAAH* AC and CC individuals (*t* (49) = 0.44, *p* = 0.659). The observed mean values of 33.4 (± 6.4, CC group) and 32.3 (± 6.5, AC group) are average compared to normative values for the German version of the STAI-T for 15- to 29-year-old men with *T*-values of 50 and 49 and percentile ranks of 49 and 45, respectively.

## Discussion

Aim of this study was to address whether the SNP rs324420 of the FAAH gene, which codes for the endocannabinoid anandamide (AEA) degrading enzyme, modulates neural and behavioral correlates of extinction recall, after successful fear conditioning and fear extinction. Carriers of the A-allele of this SNP are reported to have higher levels of circulating AEA relative to subjects homozygous for the C-allele. Between-group comparisons revealed that AC heterozygotes showed greater neural activation differences relative to CC homozygotes in core neural structures related to extinction recall, which have previously been reported in a meta-analysis [21]. While there was no clear genotype effect on fear ratings of the extinguished and unextinguished stimuli, groups differed in their state anxiety ratings, particularly after the extinction recall experiment. Trait-related anxiety group differences were not evident.

### FAAH C385A SNP group differences and neural extinction recall signaling

While we previously found no effect of genotype on neural correlates of fear extinction learning [22], contrasting the differential effect of extinction recall in the group of AC heterozygotes (*FAAH* C385A SNP) with that in the group of CC homozygotes, showed significantly greater differential neural activation in several brain regions, in accordance with prior reports [21]. Results presented here support involvement of core regions of the salience network, particularly the AIC and the ACC. From that, the group difference during extinction recall can be interpreted in a way that in the AC group greater salience network reactivity was observed upon CS^+unextinguished^ than upon CS^+extinguished^, which might represent a more successful recall of salience extinction as compared to the CC group with the reverse situation. The salience network is assumed to play a central role in emotional control and emotional processing, given its activation during the integration of sensory, cognitive and emotional input, mediation of interoceptive awareness and interaction with other networks subserving memory-related processing [28, 29]. Furthermore, a dysfunction in networks including ACC and AIC has been discussed as a potential transdiagnostic marker in psychiatric disorders [25–27]. Using a region of interest approach, Zabik et al. [19] found less amygdala activation related to the previously extinguished cue in A-allele carriers, which could be interpreted in line with our findings. However, as Zabik et al. [19] investigated trauma-exposed individuals and memory consolidation was only allowed for only 10 min after conditioning in 45 of 59 subjects, comparability of the studies remains unclear. Furthermore, while fear ratings in our study confirmed the validity of our conditioning–extinction–extinction recall paradigm [22], neither skin conductance measures nor subjective fear ratings were presented to demonstrate that the unconditioned stimulus (3D virtual snake striking toward participant's viewpoint) used was indeed suited to induce fear [19].

Following previous studies, the ACC seems to play a major role during fear extinction recall in PTSD patients, who frequently show disturbances in extinction recall and exhibit altered activation of the ACC, the hippocampus, amygdala, and ventromedial prefrontal cortex, as compared to trauma-exposed healthy controls [30–32]. In addition, AIC hyperactivity has been implied in anxiety disorders, such as generalized anxiety disorder, social phobia, and again PTSD [33, 34].

Previous human and rodent studies were able to demonstrate enhanced extinction processes in *FAAH* C385A carriers [7] given that degradation of AEA is reduced in these subjects leading to increased levels of circulating AEA. For example, mice A-allele carriers with higher levels of AEA presented decreased freezing behavior and both, human and mice A-allele carriers, displayed greater fronto-amygdala connectivity during resting-state fMRI interpreted as a biological trait marker to cope with emotionally relevant stress. Moreover, present findings are congruent with results from Mayo et al. [35], who, in one of the first FAAH inhibitor studies, found no differences in fear extinction learning between an experimental group and control group, but significant behavioral effects during extinction recall. Results were interpreted to indicate improvement of the consolidation of the learned fear extinction when AEA levels are higher [35].

Considering the effect of the *FAAH* rs324420 SNP on subjective experiences of anxiety, the present data are to be interpreted with care. While the anxiety ratings generally corroborated the validity of the experimental setup, the differences in fear ratings after the extinction recall session remained significant when comparing the extinguished and the unextinguished stimulus against the neutral stimulus. Cognitive factors might have played a role, as well as the duration of the extinction recall session, which are factors that should be addressed in a replication.

For the STAI-S, there was a significant effect of genotype indicated by the group by time interaction when taking into account numerical differences before entering the extinction recall experiment, which presumably stem from genotype-related different success of previous extinction learning. The observation of a genotype effect on state anxiety ratings from the STAI is in good accordance with previous work [7] and complements previous suggestions [7, 35], that gene-dependent elevation of AEA levels may promote fear extinction learning and recall and that neural functioning in the fronto-temporal-limbic circuit underlying extinction recall may be directly modulated by endocannabinoid signaling. These findings match with previous research conducted in rodents, where CB1 receptor agonism or inhibition of FAAH have enhanced fear extinction learning [36] and its consolidation as tested during fear extinction recall [7, 17, 37]. However, it is noteworthy to stress the importance of the time point of application as well as the individual and task-related fluctuations in the endocannabinoids, which should be subjected to future translational research [38].

### Limitations

The results are well suited to enhance the understanding of the underlying mechanisms associated with fear extinction learning. However, the study sample consisted of healthy, young, male students only, so that we were unable to determine any effects of sex and/or gender. Secondly, a transfer of the results to a clinical patient setting is limited and remains subject to future research. Next, although the sample size is rather large for an fMRI study, regarding genetic effects, a larger sample is usually required, and the results need to be interpreted with caution. Furthermore and based on previous studies, that used similar sample sizes [7], we focused solely on the *FAAH* SNP rs324420, and thus lack the possibility to control for population stratification effects that might be present in our Caucasian sample. Since these effects cannot be ruled out, a replication in a larger sample assessing more and unlinked genetic markers is important.

### Future directions

Recent reviews and studies highlight the beneficial modulatory effects of the endocannabinoid system regarding symptom alleviation and treatment of stress and anxiety disorders. Furthermore, studies have pointed out altered endocannabinoid levels in people suffering from these disorders, pointing towards a role of the endocannabinoid system not only in the treatment, but also in the development of these disorders. As recent studies have emphasized, targeting the ECS via, e.g., FAAH inhibition to enhance fear extinction learning and extinction recall seems to be a promising approach for the advancement of current psychopharmacological and psychotherapeutic treatments [35, 39, 40]. The data presented here support the potentially modulatory role of the ECS. However, more research in humans is necessary to gain a still better understanding of the underlying processes, especially before and after psychotherapeutic approaches such as exposure therapy.

## Supplementary Information

Below is the link to the electronic supplementary material.

Supplementary file1 (DOCX 35 KB)

## Data Availability

Data and material can be shared upon individual request to the authors.
