# Supplementary material for: FAAH polymorphism (rs324420) modulates extinction recall in healthy humans: an fMRI study
Source: Eur Arch Psychiatry Clin Neurosci. 2021 Dec 10;272(8):1495–504. doi: 10.1007/s00406-021-01367-4 (PMC9653364; doi:10.1007/s00406-021-01367-4)
Supplement: Supplementary file 1 — Supplementary file1 (DOCX 35 KB) [file 406_2021_1367_MOESM1_ESM.docx]

Supplementary Information

**Methods**

*Participants*

Participants were native German-speaking, Caucasian, male students without past or current history of psychiatric or neurological disorder as assessed by a preclinical Structured Clinical Interview for DSM-IV, particularly no history of any drug or alcohol abuse/dependence. We chose solely male participants for the present study to minimise confounding factors in this first translational study, since previous studies have pointed out sex differences in extinction recall ([Shvil et al. 2014](#_ENREF_4)), pain perception ([Goldstein et al. 2010](#_ENREF_2)) and stress response circuitry adaptation ([Wiesenfeld-Hallin 2005](#_ENREF_6)). From an initial sample of n = 55, data from four participants had to be excluded from final analyses due to technical failures (2), acute medical problems (1) and poor data quality (1).

Regarding lifetime exposure to cannabis, 27 of 55 participants had tried cannabis at least once previously, but no one reported regular use in the past. All participants confirmed complete abstinence from cannabis use in the two months before the experiment, and had negative THC-screening results. Further exclusion criteria were serious medical conditions, intake of any regular or acute medication (except for thyroid hormones). Participants received financial compensation of 90 €.

*Temperature calibration of the thermode*

Before the start of the main experiment, the intensity of the unconditioned stimulus (US), an unpleasant thermal stimulation on the right shinbone, was determined individually for each subject. An fMRI-compatible ATS-thermode (30 x 30 mm, TSA-II, Medoc Advanced Medical Systems, Ramat Yishai, Israel) was mounted on this location pre-treated with 0.5% capsaicin solution (Wörwag Pharma GmbH & Co, Böblingen, Germany). Intensity of the stimulation was individually determined by gradually increasing the temperature of the thermode until subjective unpleasantness of 6 out of 10 was reached on a 0 to 10 analogue scale, where 0 was "not unpleasant at all", and 10 was "the most imaginable state of pain” ([Attar et al. 2012](#_ENREF_1)). The number 6 on this scale indicated a thermal stimulation that was “clearly unpleasant, but still endurable and not painful”. This definition is in line with Lonsdorf et al. ([2017](#_ENREF_3)), who report this level as commonly used in human fear conditioning setups. Mean temperature over subjects was 46.59 °C, SD = 2.96 °C. During the main experiment, temperature oscillated between +/- 1.6 °C on average ([Spohrs et al. 2021](#_ENREF_5)).

*Experimental task during fMRI*

Three different geometric stimuli were presented on a computer screen with a duration of 4 s and an inter-trial interval (ITI) of 8-18 s (mean = 9.9 s, SD = 2.4 s), during which a fixation cross was presented on the screen. Stimulus onsets were jittered by randomly adding fractions of the fMRI repetition time.

Ramping of the US started 2.5 s after CS+ onset at a rate of 8 °C/s to achieve the maximum subjective thermal stimulation at about 4 s after trial onset. Once reaching the peak temperature, the temperature decreased, again at a rate of 8 °C/s until reaching the baseline temperature of 32 °C, resulting in a trial length of about 6 s for thermal stimulation trials.

*Pre-processing and analysis of fMRI data*

The pre-processing and statistical analyses of the MRI data were conducted using the software Statistical Parametric Mapping (SPM) 12 (r6225; Wellcome Department of Cognitive Neurology, London, UK). Data from all finally included participants met the criteria for high quality and scan stability with minimum motion (< 2 mm displacement in any one direction except for a single displacement of 2.5 mm in z-direction in one subject) and were subsequently included in fMRI analyses. The experimental EPI series were first slice time corrected and spatially realigned to their respective mean EPI volume. Afterwards, the mean EPIs and the T1 image were co-registered. Next, for normalisation to Montreal Neurological Institute (MNI) space, the T1 image was segmented using SPM’s “normalise” routine, and the resulting deformation field was applied to all pre-processed images. Subsequently, images had a voxel size of 2 x 2 x 2 mm^3^. Smoothing of the EPI volumes was performed using a Gaussian kernel with 8-mm full width at half maximum.

**References**

Attar CH, Finckh B, Buchel C (2012) The Influence of Serotonin on Fear Learning. PLoS ONE 7. https://doi.org/ARTN e42397 10.1371/journal.pone.0042397

Goldstein JM, Jerram M, Abbs B, Whitfield-Gabrieli S, Makris N (2010) Sex differences in stress response circuitry activation dependent on female hormonal cycle. The Journal of neuroscience : the official journal of the Society for Neuroscience 30: 431-8. https://doi.org/10.1523/JNEUROSCI.3021-09.2010

Lonsdorf TB, Menz MM, Andreatta M, Fullana MA, Golkar A, Haaker J, Heitland I, Hermann A, Kuhn M, Kruse O, Meir Drexler S, Meulders A, Nees F, Pittig A, Richter J, Romer S, Shiban Y, Schmitz A, Straube B, Vervliet B, Wendt J, Baas JMP, Merz CJ (2017) Don't fear 'fear conditioning': Methodological considerations for the design and analysis of studies on human fear acquisition, extinction, and return of fear. Neuroscience and Biobehavioral Reviews 77: 247-285. https://doi.org/10.1016/j.neubiorev.2017.02.026

Shvil E, Sullivan GM, Schafer S, Markowitz JC, Campeas M, Wager TD, Milad MR, Neria Y (2014) Sex differences in extinction recall in posttraumatic stress disorder: a pilot fMRI study. Neurobiology of Learning and Memory 113: 101-8. https://doi.org/10.1016/j.nlm.2014.02.003

Spohrs J, Ulrich M, Gron G, Prost M, Plener PL, Fegert JM, Bindila L, Abler B (2021) Fear extinction learning and anandamide: an fMRI study in healthy humans. Translational Psychiatry 11. https://doi.org/ARTN 161 10.1038/s41398-020-01177-7

Wiesenfeld-Hallin Z (2005) Sex differences in pain perception. Gender medicine. official journal of the Partnership for Gender-Specific Medicine at Columbia University 2: 137-45. https://doi.org/10.1016/s1550-8579(05)80042-7
